# Relying on the engagement of others: A review of the governance choices facing social media platform start-ups

**DOI:** 10.1177/02662426211050509

**Published:** 2021-12-06

**Authors:** A Rebecca Reuber, Eileen Fischer

**Affiliations:** 67131 University of Toronto, Toronto, ON, Canada; 7991 York University, Toronto, ON, Canada

**Keywords:** digital platform, platform governance, social media, user base

## Abstract

We are grateful to Professors Rebecca Reuber and Eileen Fischer for contributing our 2022 annual review article. This insightful review explores an issue of great contemporary importance regarding the relationship between entrepreneurial activities and social media platforms. Whilst there is much popular and media commentary regarding the opportunities such platforms offer for entrepreneurship, we lack informed, academic reflection upon the role and influence of such platforms for both good and ill. Hence, this review article is timely in identifying current practices and raising important issues for future research. Our thanks to the authors for their valuable contribution to the ISBJ.

Entrepreneurs create digital platforms which, in turn, facilitate entrepreneurial behaviours of others, the platform users. An important start-up activity is developing the mechanisms to govern user participation. While prior literature has provided insights on the governance of innovation platforms and exchange platforms, it has shed little light on the governance of social media platforms. In this review, we synthesize the emerging literature on diverse social media platforms, focussing on four types of governance mechanisms: those that regulate user behaviour, those related to user identification and stature, those that structure relationships among users and those that direct user attention. We highlight the implications of this body of literature for entrepreneurship scholars.

## Introduction

With advances in digital technologies, entrepreneurs are creating new organisational forms that enable business to be conducted in new ways and call into question existing theoretical frameworks (Baum and Haveman, 2020; Gulati et al., 2012). In particular, entrepreneurs are founding digital platforms to enable interactions of various kinds between disparate types of platform users (Nambisan, 2016; Nambisan et al., 2020). Digital platforms are relevant to entrepreneurship scholars because they represent new types of firms that entrepreneurs create and grow (Taeuscher and Rothe, 2020), and also because they enable entrepreneurship by others: the platform users (Cutolo and Kenney 2020; Nambisan et al., 2018).

Prior literature that provides insights on the pursuit of entrepreneurial opportunities through platform creation and via platforms created by others has focused primarily on two types: the first is innovation platforms, for autonomous suppliers developing complementary technology, such as Apple’s App Store; the second is exchange platforms, where buyers and sellers interact, such as eBay. There has been relatively little direct attention paid to social media platforms, and the scant research that has focused squarely on such businesses has been aimed at a few highly visible social media platforms, such as Facebook and Twitter. This has obscured from view the much larger number of less visible platforms. Yet, new social media platforms are continually coming into existence: Crunchbase lists over one hundred social networking ventures founded in 2020 alone. Further, social media platforms are hotbeds of entrepreneurial activity by users, with entire constellations of such users earning their living from them (Cutolo and Kenney, 2020). For example, popular users who become influencers on platforms such as Instagram and TikTok now represent a $10 billion industry (Haenlein et al., 2020). Thus, social media platforms are an important emerging phenomenon for entrepreneurship scholars to understand.

Towards this end, it is helpful to begin with definitions of both social media platforms and the user-generated content that distinguishes them. Social media platforms are interactive, scalable web and mobile technologies that allow individuals and groups to create, share, discuss and modify user-generated content (see Fischer and Reuber, 2011: 2; Kietzmann et al., 2011: 241). User-generated content refers to the text, images and sounds that users generate, circulate and interact with on social media platforms. Examples of such platforms include Facebook, LinkedIn, Pinterest, Reddit, YouTube, TikTok and Twitter. Each of these platforms has a user base of hundreds of millions of unpaid monthly active users who are continuously co-creating the value of the firm by circulating content (see Hughes, 2019). Sustaining user engagement is critical for founders of social media platforms. The size of a platform’s user base per se is not considered a key metric of success because large numbers of users are likely to become inactive, neither producing nor consuming content, without explicitly deregistering and exiting. Consistent with this tendency, the size of a social media platform’s user base is normally measured as the number of monthly active users; that is, the number of registered users who have visited or interacted with the platform over the past month (Bailey et al., 2018; Sequoia, 2019).

User behaviour on social media platforms is affected by the governance mechanisms afforded by each platform’s technologies and social structures (Bourlai, 2018). Therefore, to understand the entrepreneurial possibilities of a social media platform for both founders (the platform owner) and users, it is important to understand the choices that founders of social media platforms need to make in governing a large, engaged user base. That is the purpose of this review article. We review the emerging literature on a diverse set of social media platforms and highlight choices and constraints available to founders of social media platforms. It is important to note at the outset that the academic literature on this topic is inherently partial and dated as the real-world phenomenon is constantly evolving. Governance mechanisms are increasingly embedded in algorithms and since digital objects are continuously evolving (Kallinikos et al., 2013), they change frequently (Smith and Fischer 2020). Accordingly, in this review we highlight broad themes and draw on recent media sources more than would normally be the case.

The article is organised as followed. Firstly, we briefly summarise the literature on the governance of other kinds of digital platforms to tease out the implications of this work for founders of social media platforms. We then synthesise the literature on social media governance, focussing on four types of mechanisms: those that regulate user behaviour; those related to user identification and stature; those that structure relationships among users; and those that direct user attention. We conclude with a discussion of our findings for entrepreneurship scholars.

## Insights from research on governance of other types of digital platforms

Digital platforms have been grouped together in multiple ways (Cusumano, 2020; de Reuver et al., 2018; Vallas and Schor, 2020). Here, we identity three common types that are delineated from social media platforms – innovation platforms, exchange platforms and collaboration platforms – while recognising that the boundaries between them can be fuzzy, and that a given firm may integrate multiple types of platforms.

Innovation platforms are comprised of independent actors external to the firm who develop offerings that complement those of the digital innovation platform owner, which is usually a technology-based firm such as SAP or Oracle (Adner and Kapoor, 2010; Boudreau, 2012; Dattée, Alexy and Autio, 2018; Gawer and Cusumano, 2014; Wareham et al., 2014). This type of platform has been strongly associated with entrepreneurship in past research as the platform offers both a target market and a distribution channel for entrepreneurs – the complementors – who are delivering innovative technology-based products and can build their business on the platform (Nambisan et al., 2018). Prior research has focused on mechanisms that help to recruit complementors and keep them engaged in developing complementary offerings (Boudreau, 2012; Fang et al., 2020; Gawer and Cusumano, 2008). In addition to the need to attract a large base of customers who will purchase platform offerings (Boudreau, 2012; Rietveld and Eggers, 2018), it is important for the platform owner to strike a balance between stability, to preserve investments in complementor offerings, and evolvability, to enable complementors to adjust to changes in customer needs and technology (Wareham et al., 2014). Complementors are deterred from participation when the platform is characterised by competitive crowding, in other words, too many complementors are providing the same platform offering (Boudreau, 2012; Boudreau and Jeppesen, 2015). Indeed, platform owners selectively promote complementary products to manage overall platform value (Rietveld et al., 2019). Behaviour on the part of complementors that is unattractive to others on the platform (as well as to the platform owner itself) can be made manifest by the existence of low-quality offerings controlled by a range of standard-setting mechanisms (Wareham et al., 2014).

Exchange platforms, such as Uber, Airbnb and Etsy are comprised of independent, external actors who buy and sell products or services. This type of platform also includes crowdfunding platforms, such as Kickstarter, where external actors ‘sell’ their projects to ‘buyers’ who fund them (Mollick, 2014; Perren and Kozinets, 2018). Exchange platforms have been strongly associated with entrepreneurship in past research as they provide markets where sellers and buyers interact. As with other kinds of digital platforms, having a large base of actors on both sides of the platform attracts participation, but is insufficient to sustain it (Hagiu and Rothman, 2016). Both sellers and buyers are deterred from participating when they perceive a lack of quality among platform offerings or when they lack trust due to self-interested behaviour by some buyers and sellers that is aversive for others (Möhlmann, 2015; Mollick, 2014; Oo et al., 2019; Perren and Kozinets, 2018). Exchange platforms tend to rely on market pricing mechanisms and on trust-building features, such as reputation rating systems and dispute resolution processes, to promote positive engagement and protect against aversive behaviour (Garud et al., 2020; Hagiu and Rothman, 2016; Luca, 2017; Perren and Kozinets, 2018). Reputation rating systems constitute a governance mechanism that delegates some responsibility over governance to users. This notion of user responsibility is considered a facet of contemporary neoliberal ideology where governance is less authoritative and more market-driven; it encourages individuals to feel they can and should be responsible for their own choices and actions rather than expecting institutions to make choices for them (Shamir, 2008).

Collaboration platforms comprise of external actors who voluntarily cooperate with one another to produce a collective good; for example, the people who contribute to Wikipedia (Gallus, 2017) and Linux (O’Mahony and Ferraro, 2007). Such platforms have been less associated with entrepreneurship in past research than have innovation and exchange platforms. While the founding of the platform is entrepreneurial, and entrepreneurial behaviour such as proactiveness and innovativeness are valued in user contributions to the joint project (Von Hippel and Von Krogh, 2003), the communal value creation and capture have not been addressed in the entrepreneurship literature. Research suggests that collaboration platforms are attractive to participants when they provide opportunities to acquire valued information or skills, to solve problems that are personally or professionally relevant, to obtain recognition from others who are contributing, and to contribute to a project that is intrinsically worthy (Gallus, 2017; Shah, 2006; Von Hippel and Von Krogh, 2003). Collaborators are deterred from participating when these motivations are reduced and when there are conflicts among them (Faraj et al., 2011; Shah, 2006). Collaboration platforms sustain user engagement by providing symbolic (non-monetary) rewards that facilitate recognition (Gallus, 2017; Shah, 2006), and by instilling democratic values in governance mechanisms (O’Mahony and Ferraro, 2007). These values also tend to deter aversive user behaviours, such as those that detract from product quality and threaten property rights (Bauer et al., 2016; Gulati et al., 2012).

This examination of prior literature on digital platforms indicates that the mechanisms used to govern the behaviour of platform users vary across types of platforms. Although useful background for an examination of the governance issues of social media platforms relevant to entrepreneurship, this research stops short of shedding light on the challenges of governing social media platforms for three key reasons. First, participation on social media platforms is explicitly social in nature. Although users may be economically motivated to participate (for example, becoming an Instagram influencer, Cotter, 2019; Haenlein et al., 2020), economic gains are built on social relationships rather than through the more transactional interactions of innovation and exchange platforms. This suggests that social motivators and demotivators may be more consequential than economic ones on social media platforms, and founders need to be aware of this in enabling entrepreneurial behaviour among users. Second, users on social media platforms simultaneously adopt two roles in that they are often both producers and consumers of user-generated content. This contrasts with the other types of platforms, where user roles are distinct; users may be both sellers and buyers, for example, but not simultaneously. This means that governing an engaged user base represents a more complex challenge for entrepreneurs who found social media platforms, because any actions taken to govern user behaviour need to address content consumption and (often entrepreneurial) production at the same time. Third, by its very nature, user-generated content is not standard compared to the product listings in innovation platforms, the transaction descriptors in exchange platforms, and the work-related contributions in collaboration platforms. As a result, entrepreneurs launching social media platforms face less predictability in regard to the contributions of their users than do entrepreneurs launching other types of platforms. Thus, while providing a valuable background to our review, it cannot be assumed that this prior research addresses the governance issues in social media platforms relevant to entrepreneurs.

## Mechanisms that regulate user behaviour

### The dynamic nature of rules and regulations

Explicit rules and regulations are perhaps the most obvious tools of governance. Entrepreneurs developing social media platforms routinely develop sets of stated rules and policies that attempt to specify what users are and are not allowed to do. Typically, these rules are contained in ‘user agreements’ or ‘terms of service’ to which users must consent when they create an account. While behaviour that is barred varies between platforms and within platforms over time and across regions, virtually all platforms specify certain types of prohibited content. For example, as of July 2021, Reddit rules are relatively few in number. Its policies stipulate only that:

Reddit is a place for creating community and belonging, not for attacking marginalised or vulnerable groups of people. Everyone has a right to use Reddit free of harassment, bullying, and threats of violence. Communities and users that incite violence or that promote hate based on vulnerability will be banned. (https://www.redditinc.com/policies/content-policy)

Instagram’s policies, by contrast, are more elaborate as the following excerpts from its 2021 policy indicate:

Instagram is not a place to support or praise terrorism, organised crime, or hate groups. … We want to foster a positive, diverse community. We remove content that contains credible threats or hate speech, content that targets private individuals to degrade or shame them, personal information meant to blackmail or harass someone, and repeated unwanted messages… We have zero tolerance when it comes to sharing sexual content involving minors or threatening to post intimate images of others.

It’s never OK to encourage violence or attack anyone based on their race, ethnicity, national origin, sex, gender, gender identity, sexual orientation, religious affiliation, disabilities, or diseases. When hate speech is being shared to challenge it or to raise awareness, we may allow it. In those instances, we ask that you express your intent clearly.

Serious threats of harm to public and personal safety aren't allowed. This includes specific threats of physical harm as well as threats of theft, vandalism, and other financial harm. We carefully review reports of threats and consider many things when determining whether a threat is credible…. (https://help.instagram.com/477434105621119?ref=igtos)

On one hand, a relatively limited set of formal rules to try to enforce, as in the case of Reddit, can make detection of infractions more manageable and facilitate the freedom of speech, deeply entrenched in the values of societies such as that in the U.S. Having a greater range of rules and prohibitions, on the other hand, can empower the platform to take down a wider range of content that is offensive to some users, and may diffuse some criticisms that the platform is fostering uncivil and manipulative behaviours. However, perceived failure to enforce stated policies can exacerbate such criticisms, and perceived excessive enforcement can likewise do so (Rainie et al., 2017).

Establishing rules and regulations is a dynamic activity for founders of social media platforms, because existing rules and regulations are subject to frequent contestation. Sometimes this contestation is due to dynamics particular to the firms, such as new user behaviours emerging on the platform. Twitter, for example, notes that its ‘Twitter Rules’ are a living document for this reason (Harvey, 2018). Sometimes contestation is due to systemic events, such as the COVID-19 pandemic which created new challenges for governing user-generated content (Baker et al., 2020). For example, a group of subreddit moderators wrote an open letter to Reddit calling on company administrators to remove misinformation and disinformation on COVID-19 (Porterfield, 2021). Contestation of existing rules can also be a result of the increasing internationalisation of a social media platform which is accompanied by the need to conform with local laws, such as Germany’s law banning online hate speech (Saurwein and Spencer-Smith, 2020).

Entrepreneurs creating rules and regulations to govern user behaviour on their platforms must recognise that the nature of the initial rules (or lack thereof) sends a significant signal to users and will attract some while alienating others. Consider the example of Parler, which drew in its initial user base by setting minimalistic restrictions on user behaviour, thereby attracting users away from platforms with more elaborate rules, but which also earned it a reputation for fostering violence. At the same time, entrepreneurs must take into account the considerable costs associated with updating rules to deal with internal dynamics, systemic events and/or external pressures. External pressures are likely to increase as platforms grow, since larger organisations in general have greater public visibility and are more likely to attract the attention of the state, the media, professional groups (Luoma and Goodstein, 1999). In other words, as the size of a social media platform’s user base grows, the entrepreneurs who run it will need to devote increasing resources to monitoring the appropriateness of its current rules, and even more to enforcing them. This suggests that, relative to other types of startups, entrepreneurs who start social media platforms will need to invest early on in managing the public policy implications of their company.

### Content moderation

Enforcing content-related rules inevitably requires interpretation and involves ‘content moderation’, which refers to the organised practice of screening user-generated content in order to determine the appropriateness of the content for a given site, locality or jurisdiction (Roberts, 2014). Those managing social media platforms may deploy some combination of three main approaches to content moderation: algorithms powered by artificial intelligence (hereafter algorithmic moderation); paid, trained, human content moderators; and unpaid peer users (Schwarz, 2019).

Algorithmic moderation entails systems that ‘classify user-generated content based on either matching or prediction, leading to a decision and governance outcome (e.g. removal, geoblocking, account takedown)’ (Gorwa et al., 2020: 3). While such algorithms have been credited with significantly improving the ability of a platform to detect user-generated content that violates their rules and regulations, they are far from being sufficient for all governance purposes. They are particularly poor at detecting nuances and contextual variations present in human speech, and routinely fail to identify hate speech and misinformation, or may do so erroneously (Gorwa et al., 2020; Singh 2019).

Given the limitations of algorithmic moderation, owners of social media platforms may also enlist the services of trained, paid content moderators to assist with enforcing rules and regulations (Singh, 2019). These content moderators are given detailed flowcharts and decision trees to follow when flagged content is forwarded to them. In some cases, they have full discretion in regard to whether or not to remove or label content; in others, that decision ultimately rests with platform managers (Roberts, 2014). A third mechanism to enforce standards is the use of ordinary, unpaid platforms users, rather than trained, paid content moderators. This relies on affording responsibility to users (Shamir, 2008), similar to the rating and review systems of exchange platforms. Social media platforms encourage users to report content they believe is inappropriate or offensive. Complainants typically must choose the alleged offense from a list, and often cannot add details, context or explanations (Schwarz, 2019). Once users report concerns, paid content moderators and/or platform administrators make determinations as to whether the flagged content violates platform policies, and what if any action should be taken to enforce platform rules.

There are benefits and drawback to all these approaches that entrepreneurs need to be aware of in founding social media platforms, and entrepreneurship scholars need to be aware of in studying the growth and impact of social media platforms. The benefit of algorithmic approaches is that they are scalable, in that as the content on a platform proliferates, algorithms can efficiently handle larger volumes. Moreover, algorithmic moderation can lead to rapid detection and take down of materials that violate platform rules, and algorithms can be readily updated to reflect new rules or new means devised by users to evade them (Gorwa et al., 2020). The drawback of algorithmic moderation is that machine learning systems are notoriously poor at making nuanced, context-dependent judgements (Duarte et al., 2017). There is widespread civil society concern that automated systems lead to ‘overblocking’ that undermines freedom of speech, as well as to unwitting but pervasive systemic biases against marginalised groups (Binns, 2018; Witt, 2020).

There are likewise benefits and drawbacks to the use of paid, trained, content moderators. On the plus side, these moderators are in principle, capable of making nuanced decisions that are more appropriate than those rendered by algorithms. At the same time, content moderators are not immune to systemic biases (Witt, 2020). Moreover, the work of content moderation is controversial given the toll it exacts on those paid to do it. As Roberts (2014: ii) notes, paid content moderators are exposed to ‘images and material that can be violent, disturbing and, at worst, psychologically damaging’. Engaging unpaid users in content moderation has the benefit of letting users feel empowered to shape the platform according to their own principles and tastes. Moreover, user input into content moderation on social media platforms can be an ‘important and necessary part of maintaining user-friendly spaces’ as when users become ‘a volunteer corps of regulators’ (Crawford and Gillespie, 2016: 411). A drawback of user engagement in content moderation is that users may be disappointed if their complaints do not result in the governance responses for which they had hoped: as a result, they may become disaffected from platforms and engage in negative word of mouth as a result. This is increasingly likely to happen as social media platforms grow and their user base becomes more heterogeneous.

Founders of social media platforms may initially rely on only one of these governance mechanisms; however, as the platform grows, it is likely that some combination of all three will be used to meet the mounting pressures imposed by external stakeholders (Singh, 2019). To be effective at using algorithmic moderation, entrepreneurs will need to make significant ongoing investments in personnel with relevant computing and data analytic skills. The expenses required to pay human content moderators will increase as the platform attracts engaged users, and no economies of scale are associated with this mechanism. As an extreme example because of its size, Facebook hires more than 15,000 people and 10 firms to moderate its posts manually: it is estimated that the consulting firm Accenture alone receives $500 million per year for this activity (Satariano and Isaac, 2021). It has also had to provide compensation to content moderators for mental health issues arising from their job (Newton, 2020). Entrepreneurs, and entrepreneurship scholars, also need to understand the social, as well as the financial, costs of this human content moderation to understand the impact of social media platforms (see Chen, 2014; Satariano and Isaac, 2021). Entrepreneurs will need to have thought through these issues – and the resources it might take to manage objectionable content – when approaching investors, because investors will know that setting, updating and enforcing content-related rules is a core challenge facing all social media platforms.

An additional consideration for entrepreneurs is that aversive platform content may limit opportunities. This was observed when the violent content on Parler led the platform to be suspended from the app stores of Apple and Google and from Amazon’s cloud services (Schoolov, 2021). Exit opportunities may also be constrained because failure to implement effective rules regarding user-generated content may make potential buyers wary. For example, Disney and Salesforce dropped their bids for Twitter in 2016 because of the platform’s reputation for failing to enact and enforce rules to restrict objectionable content (Ingram, 2016). At the same time, however, we note that platforms with persistent reputations for content moderation problems can attain substantial valuations, as is evident in the case of Facebook (Su, 2021). This suggests that although regulating user behaviour is an important challenge for founders of social media platforms, it does not preclude such platforms from being significant entrepreneurial opportunities.

## Mechanisms related to user identification and stature

Beyond rules and regulations, a fundamental choice for entrepreneurs designing the governance of a social media platform is whether and how users will be identified. When users have a persistent identity on the platform, their contributions to the platform can be recognised and they can gain stature on the platform, but when they are anonymous, this cannot happen. The governance mechanisms relevant to both user identification and stature are discussed in this section.

### User identification

The biggest divide related to user identification among social media platforms is whether a user’s identity is linked to their real-world identity or whether it can be linked to a pseudonymous identity. Examples of platforms based on real-world identities are Facebook, Instagram, LinkedIn, Nextdoor and YouTube. Examples of platforms based on pseudonyms (which can signal real-world identities if a user chooses a pseudonym that discloses their real identity) are Pinterest, Reddit, TikTok, Tumblr and Twitter. Founders of social media platforms may opt to insist on real identities for several reasons. One is transparency: there is a direct link between the posted content and a human user. Facebook attempts to enforce a ‘real name policy to heighten safety and accountability, as well as to target engagement and advertising tactics (Haimson and Hoffmann, 2016). The disadvantage of this being that people who fear public visibility and/or online harassment may be reticent to join the platform. A second reason for insisting on real identities is that they can allow the platform owner – or the existing platform users – to screen out certain types of users. For example, ASmallWorld comprises a small exclusive social network of celebrities, where a new person can join only if invited by existing members who have been granted inviting privileges (Papacharissi, 2009). Nextdoor links user accounts to a confirmed residency in a neighbourhood. Screening out potential users and partitioning the user base by neighbourhood can lead to homogeneity among users and focused content and discussions highly relevant to those users. Disclosing real identities can lead to the development of fruitful offline interactions, but the downside of this is that it can also lead to online and physical surveillance and threatening behaviour (Kurwa, 2019; Nagle, 2017).

A pseudonymous platform also has benefits and drawbacks that entrepreneurs need to consider when they design a platform, and entrepreneurship scholars need to consider when studying them. Pseudonyms are user identifiers that may or not reflect a user’s real identity. Pseudonymity enables users to share ideas and content without worrying that they will become publicly identified (Kesvani, 2019). This may be because they fear visibility and/or because they view their real authentic identity as different than that they present to the world. Twitter, for example, has long-defended pseudonymity as a way to enable self-expression and free speech under regimes of censorship and repression, such as during the Arab Spring. Similarly, Tumblr has been seen as a safe space to explore gender fluidity and intersectionality (Nagle, 2017). Renninger (2015: 1519) asserts that while Facebook is ‘more closely tied to the maintenance of a true identity often tied to a real name’, pseudonymity facilitates counterpublic communication and enables the sharing of countercultural ideas. At the same time, pseudonymity renders it easier to post content that is offensive to other people without the threat that it will be linked to a real person.

Whether or not a platform requires real identities to be confirmed and disclosed, users often try to impersonate famous people, by fraudulently claiming their identity. As a result, social media platforms began to ‘verify’ the accounts of those they believed to be most vulnerable to impersonation. These are people ‘of high public interest’ (Twitter) or a ‘well-known, highly searched for individual, brand or entity’ (Facebook). The verification is publicly visible; for example, Twitter, Facebook, Instagram, YouTube and TikTok place a blue check mark beside an account holder’s handle to signify that the account has been verified. The high adoption rate of this governance mechanism across social media platforms suggests it is one that founders may wish to plan for from start-up.

After having considered the use of real identities and pseudonyms, we now consider the much rarer occurrence of platforms where identities are completely anonymous, with no track record. The most widely known example is 4chan, where the default identity is Anonymous (Dibbell, 2010). An anonymous identity allows users to post whatever they wish without consequences; 4chan’s founder has said that anonymity fosters creativity because users are not afraid to fail in their postings and are judged on their ideas rather than who their identity (Dibbell, 2010). Yet, anonymity and a lack of consequences also results in content that is widely viewed to be racist, homophobic, misogynistic and cruel (Nagle, 2017). For example, discussing the enormous amount of online abuse that happens on social media platforms, Twitter founder Evan Williams stated: ‘You can participate under a pseudonym or something, but there needs to be longevity to, and a history of, your actions. So, there has to be a cost to abandoning an identity and creating a new one, because if there is not, then there are no consequences for acting badly, as there is in society’ (Aspen Institute, 2011). This cost needs to be assessed by founders who are considering allowing anonymity among users because it can have a substantial impact on the reputation, and the social impact, of a social media platform as its user base grows.

### User stature and influence

When users have a constant identity on a social media platform – whether it is their real identity or a pseudonym – the platform owner can govern their behaviour by providing ways for them to acquire stature and influence on the platform. This can be undertaken through social endorsements that adhere to user identity and through leadership roles that the platform owner enables. In general, when entrepreneurs enable users to gain visibility and stature on a platform, they are providing a means for entrepreneurial users to pursue their own opportunities on the platform. An immediately visible signal of stature on some social media platforms is a user’s network size: a count of the number of users associated with a focal user. Disclosure mechanisms vary: on LinkedIn, the disclosed network size is capped at 500, and on Facebook, a user can choose to hide their network size. Highlighting a network size can attract and retain users because it signals reputation to both the online and offline worlds, enhancing a user’s ability to monetize their participation on the platform. It also provides a disincentive to leave the platform and start building a network elsewhere.

A user’s stature on a social media platform can also be created through social endorsements of the content the user has posted. For example, on Reddit, users have a karma and a longevity score. A high karma score indicates both that someone is active on Reddit and that they post content valued by other users: it is calculated through the ‘upvotes’ and ‘downvotes’ that others make on their posts, as well as the comments they make on threads. A longevity score indicates how long a user has been a registered as a Reddit user. Given the norms of pseudonymity on Reddit and the difficulty of becoming influential through offline activities (which is possible for certain users of other platforms such as Twitter and Instagram), these scores would seem to have the potential to signify online opinion leaders, although the empirical evidence for this is scant (Kilgo et al., 2016).

Even when a user’s stature or ‘influence’ on a social media site is affected by the behaviour of other users, the underlying algorithm is managed by the platform. In developing these algorithms, the platform owner is deciding which users and which content are recognised and how. Entrepreneurs as platform owners exercise governance through their operationalizations of abstract concepts like ‘content users care about the most’ and ‘meaningful social interactions’. They also need to decide how transparent these algorithms should be, because users will attempt to ‘game’ the algorithm to increase their stature on the platform (Cotter, 2019). In addition to visible metrics, users can acquire stature and influence on a social media platform through playing a leadership role on the platform. This mechanism is perhaps most visible on Reddit. Any user can create a virtual community (a subreddit) and become the content moderator of that subreddit, able to delete posts they feel violate the community’s standards. Across Reddit the removal of posts is fairly frequent: a recent study found that over 20% of Reddit posts were removed during a six-month period in 2018 (Jhaver et al., 2019). However, moderation across the thousands of subreddits varies widely. While some subreddit moderators have written rules – for example, suggesting why users might ‘upvote’ or ‘downvote’ a particular post – such rules are not always enforced (Julien, 2019). Distributed moderation will appeal to founders who wish to encourage heterogeneous virtual communities with their own rules and norms, but not to founders who prioritise centralised and standardised rules and norms across the platform. Finally, as has been noted, anonymity prohibits users from developing a stable reputation or stature on the platform (Grimmelman, 2015). Trammel reports that a user can attempt to do this on 4chan, by ‘tripping’ with a unique code that identifies a specific content poster. However, users who do this are often attacked by other users as ‘craving attention and possessing an undue sense of self-importance’ (Trammell, 2014: 4). This suggests that while anonymity can support consequence-free norm violation, it is not necessarily associated with user autonomy. Although it seems inconsistent, it appears that user anonymity may result in a strong collective code enforced by the user community (Grimmelman, 2015; Huang and Li, 2016; Nagle, 2017). The literature on 4chan suggests that user anonymity as a platform feature is one that becomes deeply ingrained into the platform’s culture. This reinforces the caution that anonymity is a feature that entrepreneurs need to consider very carefully before adopting.

## Mechanisms that structure relationships among users

Many social media platforms provide features which allow users to link themselves with other users and track their activity on the platform. These, too, serve as governance mechanisms encouraging some forms of user behaviour and discouraging others, and so are important to understand for founders and entrepreneurship scholars studying social media platforms. Platforms can be designed such that links must be symmetrical, such as Facebook friends and LinkedIn contacts; in such cases, users have ‘gated access to other users. Alternatively, platforms can allow asymmetrical relationships; this is exemplified by Digg and Snapchat friends, and Twitter, TikTok and Instagram followers. For founders wanting to enable the pursuit of entrepreneurial opportunities by users, asymmetrical relationships are better able to allow users to amass a group of followers. For example, sports and entertainment celebrities can acquire a network of online fans without having to follow them in return. A third linking option, followed by Snapchat and Pinterest, allows users to specify a small network (one or two other users) to view a specific item of posted content. This mechanism is of interest to founders who wish to attract, and retain the engagement of, users who are most interested in fostering personal collections and connections, rather than developing a wider social net (Lui, 2015). Social interaction is not limited to connections between dyads of users. Some social media platforms have features promoting virtual communities around share interests, such as Tumblr fandoms, Reddit subreddits and Facebook groups. These can have specialised, in-group jargon (Bourlai, 2018). Entrepreneurs wishing to develop virtual communities on their social media platform in order to try to bind users to the platform should pay attention to three social affordances related to such communities: regular engagement, personalised profiles and features facilitating social connections and responses to content (Parks, 2011: 108).

Many social media platforms enable users to respond to the content of other users. One type of response is endorsement; for example, Facebook and Instagram likes and Reddit’s upvotes and downvotes. Another type of mechanism is sharing a user’s content; for example, through retweets (Twitter) or reblogs (Tumblr). As discussed in the previous section, both mechanisms make the originator of the content more visible and can contribute to their stature on the platform. Sharing a contribution – distributing it more widely through one’s own network on the platform – increases its visibility and reach. This is beneficial if it is content that encourages users to stay engaged with the platform, but a drawback if it is content that users find distasteful and discourages their engagement. Since sharing content is based on networks on a platform, it can heighten the effect of the choices entrepreneurs make with respect to the types of networks that are supported on their platforms. Further, if users are enabled to pass content along, they may be able to respond to it more actively, such as the comments that are allowed with retweets in Twitter. Tumblr expressly does not allow users to comment directly on reblogs, a constraint which the platform’s founder believed would reduce hostile comments; instead, users have to reblog the original content with their own content attached (Ringrose and Lawrence, 2018). Another comparison in this regard is TikTok and YouTube. TikTok users can indicate that their content is private, to protect it, or allow other users can interact with it. TikTok provides features enabling interaction, such as the duet function, that lets users create a video based on a previously created video, with both running together. In contrast, YouTube’s platform does not facilitate the generation of content from posted content: there is no built-in way to reuse videos created by other users or to let users reuse one’s own videos (Burgess et al., 2008). Such liberties and constraints on sharing content constitute part of the core functionality of social media platforms, and are therefore part of the value proposition that entrepreneurs offer users through their platform.

Papacharissi (2009) draws on Goffman’s (1963) notion of tight and loose social situations to distinguish between governance mechanisms that foster tighter and looser interactions between users. She shows that LinkedIn is comprised of mechanisms that foster relatively tightly scripted interactions, whereas the interactions possible on Facebook are much looser. That said, Facebook provides tools for users so they can tighten up their connections with other users if they so wish. Neither tighter nor looser interactions are inherently better or worse, but they provide different value propositions, and entrepreneurs should decide which best suits their target market. Looser interactions enable ‘taste performances’ on the part of users to a greater extent, whereas tightly scripted interactions foster conformance with platform-wide norms (Papacharissi, 2009).

## Mechanisms that direct user attention

A fourth category of governance mechanisms available to entrepreneurs designing social media platforms is those that direct user attention toward, or away from, certain content. These mechanisms are critical both for ensuring users find valuable content that maintains their interest in that platform and for preventing them from engaging in actions that are undesirable from the perspective of the platform owner and/or civil society. Mechanisms that direct user attention include features that; facilitate searches; curate content; and, educate users about the content to which they are being exposed.

### Features that facilitate search

When platforms are new and small, initial users may have common interests and may therefore have little difficulty finding content of interest to them. However, platforms need to grow their engaged user base to be successful. Two consequences of growth in the engaged user base are that the volume of content grows, and the user base becomes less homogenous. The combined result of these consequences being that users need search features to help them find the content in which they are interested. Striking evidence of this need on the part of users can be found in the history of Twitter: the hashtag (#) feature that allows people to tag content so that others can search for it was created by an early adopter of the Twitter platform, Chris Messina, out of his frustration that ‘there was no way of organizing tweets so you knew what to pay attention to and what to ignore’. (Pandell, 2017).

Features that enable users to help others to find their content (such as hashtags [Instagram and Twitter]) and keywords [LinkedIn] and even emojis [Snapchat]), and those that allow users to seek out specific content of interest to them, are now incorporated in virtually every social media platform. Of course, founders of social media platforms do not rely solely on users to tag their own content. Rather, they provide search technologies that scan the content of posts to find material relevant for a user who executes a search. They refine the governance impact of these search features by customising the results of the search depending on user characteristics. For example, LinkedIn explains to users that they deploy ‘proprietary algorithms’ that they are continuously updating in order to ‘optimise’ search results. They state that

‘Before we return results, we consider the searcher’s activity on LinkedIn, the profiles returned by the query, and other members who have run similar searches in determining the sort order. We also consider your search history to predict results that are likely to be more relevant to you. These, along with other factors, combine to provide us with data to improve the overall quality of our members' search results’. https://www.linkedin.com/help/linkedin/answer/4447/linkedin-search-relevance-people-search?lang=en

LinkedIn is by no means unique it attempting to ‘optimise’ search, a practice that in effect means only a subset of possible results are presented in response to a search. Indeed, it is critical that entrepreneurs developing social media platforms anticipate the need to invest in developing and updating algorithms that ensure user searches result in relevant content appearing high in the list of results. If too much irrelevant content clutters search results, user attention can be lost, and their engagement diminished.

### Features that curate content

The term ‘curating content’ refers to identifying content of relevance to users and drawing it to their attention; it also refers to limiting the chances users are exposed to content that may be aversive or harmful. While search features give users tools to seek out content themselves, features that curate content attempt to expose users to content that is likely to attract their attention and be of value to them but for which they themselves did not search. One way that entrepreneurs offer this facility is by enabling the platform to make suggestions to users. For example, Twitter identifies trending topics on the platform and when users visit the platform, they can choose to click on a trend that interests them. YouTube makes recommendations about what video to watch next in the bar beside the video currently showing. Instagram recommends posts from accounts users do not already follow. And TikTok provides a ‘For You’ feed to bring to attention content they believe will hold the user’s interest.

Content is also curated by algorithm-driven features that ‘uprank’ and ‘downrank’ elements in the stream of content that a user sees. For example, Facebook has used a ‘news ecosystem quality’ (NEQ) score to uprank certain news sources over others. Instagram uses machine learning to determine what the order of photos and videos in a users’ feed will be, based on the likelihood they will be interested in the content, their relationship with the person posting and the timeliness of the post. Conversely, downranking can reduce the number of times content appears in other users’ social media feeds, often algorithmically. For instance, Facebook downranks ‘exaggerated or sensational health claims, as well as those trying to sell products or services based on health-related claims’. At the extreme end of this spectrum, content may even be downranked to 0, or ‘no ranking’, meaning that content remains on the platform, but it is not algorithmically delivered to other users in their feeds (Saltz and Leibowitz, 2021). ‘Shadowbanning’ is one variant of such downranking in which user posts are hidden from other users (Are, 2021; Saltz and Leibowicz, 2021). Collectively, these forms of content curation are critical tools in the arsenal of entrepreneurs designing and growing social media platforms. Making relevant content more immediately visible reduces the chances of losing user attention. Downranking content that is either less interesting to the user, or potentially harmful to them or to society, reduces the risk that the platform will alienate users or incur sanctions from external stakeholders.

### Features that educate users about content

A contemporary approach to governing the attention of users takes the form of adding some information that gives context to a post or stream of posts. It was relatively uncommon until 2016 when Facebook, under pressure to curb fake news on the platform, began to partner with third-party fact-checkers at media outlets (Silverman, 2016). Entrepreneurs who use this family of features on their social media platform are generally aiming to diminish attention to information that lacks credibility. This is a dynamic category of governance mechanisms, with new approaches being introduced with great frequency (Saltz and Leibowitz, 2021).

Labeling individual posts is one common approach within this family of mechanisms. ‘Veracity’ labels are direct corrections to inaccurate posts, informing readers that information is disputed, whereas ‘contextual’ labels provide additional information about posts, such as their sources or who paid for them (Morrow et al., 2020). For example, in 2020, Twitter introduced a new practice to label ‘information’ that had been ‘significantly and deceptively altered or fabricated’ (Roth and Acuthan, 2020). When tweets were thus labelled, users were warned before they were allowed to retweet or like the tweet, and, in some cases, additional explanations or clarifications were added such as a landing page with more context. As another example, TikTok uses fact checkers to verify claims made in user videos. In 2021, it implemented a practice of flagging unsubstantiated content using a banner saying the content had been reviewed but could not be conclusively validated. If a user tries to share a flagged video, they receive a prompt that reminds them the video has been flagged as unverified, to give that user a moment to pause and reconsider their actions (Perez, 2021). Beyond labeling individual posts, entrepreneurs can also decide to provide information about an account or a group of accounts on a social media platform. This information may include details such as the account’s country of origin or its sponsor. For example, YouTube provides information that indicates whether an account is state sponsored. As another example, Twitter, Instagram and Facebook linked posts to credible election-related websites prior to the 2020 US election (Saltz and Leibowitz, 2021).

These educational approaches have increased in lockstep with the widespread criticism of the harmful societal impact of social media platforms. Such approaches can be costly for founders of social media platforms to implement and sustain, and the efficacy of specific practices is currently unclear (Morrow et al., 2020). That said, entrepreneurs concerned with striking a balance between enabling free speech and protecting users from misinformation and other harmful content are likely to need to invest in educating users about the content posted on their platform.

## Discussion

Social media platforms are a new type of organisation whose value is created, to a substantial extent, by autonomous individuals only loosely bound to it. Accordingly, entrepreneurs founding social media platforms face governance challenges that are very different from that faced by those who need to focus mainly on employment and partnership relationships. These challenges are greater than those of other types of digital platforms because the content is inherently social, rather than transaction-based or work-based. It is not surprising that most of the public debate over the content of digital platforms focuses on the governance of social media platforms. Further, the contestations on social media platforms, and the approaches for dealing with them, are constantly changing as users learn how to use platforms to advance their own agenda. As the foregoing sections imply, for entrepreneurs there are no sure-fire, tried-and-true recipes for success.

Although the entrepreneurs who found social media platforms may start with a focused value proposition and target market, these are likely to broaden as they grow its user base, resulting in greater heterogeneity of that user base. Guardrails and guidelines that worked initially may diminish in effectiveness. And, the time span over which this happens may be decreasing in length: as people have become more familiar with social media platforms, the initial ‘honeymoon’ stage of having little offensive content is becoming shorter. For example, Roose (2021) reports that within 11 months after start-up, Clubhouse – a social media app based around voice chat rooms – had more than 10 million users and a valuation of $1 billion. Yet, within weeks of start-up, it was facing accusations of enabling hate speech, even though access to the platform is by invitation only.

What does this mean for entrepreneurship scholars? There are several interesting directions for possible research on the founders and founding of social media platforms. While we have evidence that entrepreneurs are using social media platforms to develop their own non-platform ventures (Fischer and Reuber, 2011, 2014), we do not have systematic accounts of the processes involved in starting a social media venture. As with other kinds of ventures, there is some indication that founder experiences and preferences persist even with substantial user growth. For example, Twitter’s initial design reflects its founder’s background in blogging and SMS (Bilton, 2013) and the resulting 140-character limit lasted a decade. Pinterest’s initial images were chosen to correspond with the founder’s tastes, and the same aesthetic was observed on the platform five years later (Lui, 2015). It would be valuable to conduct a longitudinal study of a panel of very new social media platforms, founded in the same temporal cohort, to examine the persistence of governance mechanisms as the user base grows and diversifies, and as user behaviours and pressures from stakeholders evolve. Cross-sectionally, such a panel could be the basis of developing a typology of social media platforms in terms of the affordances they provide users, their early adopters, and the governance challenges that ensue. Related, the challenge of aversive online content is fundamental to social media platforms, and future research could examine the financial, social and reputational costs in managing it, and the rationale for the trade-offs that are made in different circumstances.

Another opportunity for future research relates to users-as-entrepreneurs using social media platforms to pursue their own opportunities. Many platforms have flourished in part because users can derive an income from participating in them (Smith and Fischer, 2020). This is increasingly true of social media platforms, as they start to provide directly monetizable entrepreneurial opportunities for users. As has been discussed in this review, certain governance mechanisms related to user identification, user stature, platform-based networks and content selection and creation can increase entrepreneurial possibilities for users. As well, we expect there to be more special-purpose facilities to promote entrepreneurship on social media platforms. For example, Twitter recently launched a Super Follows facility where people can earn revenue by sharing subscriber-only content with their Twitter followers (Crawford, 2021). A signal that this type of activity is likely to grow is the development of an investment ecosystem to support social media content creators (Lorenz, 2021).

As has been noted in regard to innovation and exchange platforms: ‘the power asymmetry at the heart of the relationship between the platform and its ecosystem members is intrinsic to the economics and the technological architecture of digital platforms [and] the conditions of engagement for platform entrepreneurs are so different from traditional entrepreneurship that these entrepreneurs are more usefully termed “platform-dependent entrepreneurs”’ (Cutolo and Kenney, 2020: 1). While it seems obvious that this power asymmetry is also true for entrepreneurs who are dependent on social media platforms (Caplan and Gillespie, 2020), what is less clear is how social media platforms should manage their relationships with platform-dependent entrepreneurs who, collectively, provide content that is critical to attracting other users. In particular, it is unclear when and how platforms should add financial governance mechanisms to others that have been reviewed in this article; entrepreneurship scholars could provide value by exploring this topic.

Research that takes the perspective of entrepreneurs who depend on social media platforms is also required. Those who take advantage of opportunities created by exchange and innovation platforms frequently engage in ‘multi-homing’, that is, operating on several platforms at once (Cutolo and Kenney, 2020). User entrepreneurs are aware of how algorithmic governance mechanisms, such as shadow banning on Instagram, affect their visibility and monetization possibilities (Are, 2021; Cotter, 2019). Entrepreneurship scholars can build on the tendency of user-entrepreneurs to operate on multiple platforms to explore how they diversify their monetizable content and risks by operating on different social media platforms, and how the governance practices of social media platforms influence this practice.

Finally, it is important to future entrepreneurship research on social media platforms for scholars to go beyond studying the established, highly visible, platforms such as Facebook, Instagram, YouTube and Twitter. While these are important cases, they are not fully representative of the entire category: they have path dependencies originating from their points of origin, and because of their size they are subject to heightened scrutiny from the media, from governments, and from citizens. New start-ups adopting this organisational form will differ in important ways from these ‘senior citizens’ of the social media landscape. While our review has pointed out some rudimentary elements of social media governance problems and practices by looking at established platforms, it is vital that future research look at emerging platforms and the objectives and priorities of their founders and how these are encapsulated in the way they govern user engagement.
